# Variations of collagen-encoding genes are associated with exercise-induced muscle damage

**DOI:** 10.1152/physiolgenomics.00145.2017

**Published:** 2018-05-25

**Authors:** P. Baumert, G-REX Consortium, C. E. Stewart, M. J. Lake, B. Drust, R. M. Erskine

**Affiliations:** ^1^Research Institute for Sport & Exercise Sciences, Liverpool John Moores University, Liverpool, United Kingdom; ^2^Institute of Sport, Exercise & Health, University College London, London, United Kingdom

**Keywords:** *COL1A1* single nucleotide polymorphism (SNP), *COL5A1* gene, delayed-onset muscle soreness (DOMS) phenotype, exercise-induced muscle damage (EIMD), lateral force transmission

## Abstract

We investigated whether single nucleotide polymorphisms (SNPs) within genes encoding the alpha-1 chain of type I (*COL1A1*, rs2249492; rs1800012), type II (*COL2A1*, rs2070739), and type V *(COL5A1*, rs12722) collagen were associated with the variable response to exercise-induced muscle damage (EIMD). Knee extensor muscle strength and soreness were assessed pre-, post-, and 48 h post-EIMD (120 maximal eccentric knee extensor contractions) in 65 young healthy participants, who were genotyped for the aforementioned SNPs. We found that *COL1A1* (minor) T-allele carriers (rs1800012) and (major) T-allele homozygotes (rs2249492) were generally weaker (*P* ≤ 0.019); and (minor) A-allele carriers of *COL2A1* (*P* = 0.002) and (major) T-allele carriers of *COL5A1* (*P* = 0.004) SNPs reported greater muscle soreness, all compared with their respective major (rs1800012; rs2070739) and minor (rs2249492; rs12722) allele homozygote counterparts. To conclude, the risk alleles of these four SNPs appear to negatively influence muscle strength and post-EIMD recovery, possibly via a dysregulated collagen network affecting the muscle’s mechanical properties.

## BACKGROUND/MOTIVATION FOR THE STUDY

Unaccustomed strenuous exercise involving eccentric muscle contractions (i.e., when the muscle is active while being forcibly lengthened) induces ultrastructural muscle damage. However, the changes in muscle damage-related biomarkers, e.g., loss of strength and delayed-onset muscle soreness, differ considerably between people (1).

Tendon and the extracellular matrix (ECM), which surrounds the contractile elements of the muscle, are composed of different types of collagen. These influence the mechanical properties of the muscle-tendon unit (MTU), ultimately influencing the transmission of force from the muscle to the bone. The ECM enables muscle force to be transmitted both longitudinally and laterally to the tendon, and, during eccentric contractions, external force can be transmitted laterally to the sarcomeres, causing MTU damage (5).

Single nucleotide polymorphisms (SNPs) of collagen genes have been associated with MTU mechanical properties (4) and injury risk (2), possibly by affecting RNA stability and hence collagen abundance. Furthermore, resistance exercise increases collagen gene expression (3), but no study has investigated whether SNPs of collagen-encoding genes are associated with the response to exercise-induced muscle damage (EIMD). Therefore, we aimed to investigate if intron SNPs within the *COL1A1* (*T/C*, rs2249492; G/T rs1800012) and in *COL5A1* (T/C, rs12722) genes, and a missense SNP within the *COL2A1* (G/A, rs2070739) gene, which encode the alpha-1 chain of type I, V and II collagens, respectively, were associated with the skeletal muscle response to EIMD in untrained young men and women.

## PHENOTYPE

Following familiarization, all participants performed the following assessments before, immediately after, and 48 h after, 12 sets of 10 maximal eccentric unilateral knee extension contractions (EIMD protocol): *1*) maximum voluntary isometric (80° knee flexion) and *2*) isokinetic (60°/s) knee-extension contraction (MVC) torque (Humac Norm; CSI, Stoughton, MA, and Biodex Multi-Joint System 3 Pro, Shirley, NY) normalized to body mass; and *3*) muscle soreness [visual analog scale (0–10 cm; 0 cm = no soreness; 10 cm = maximal soreness) in conjunction with a three-repetition squat]. A venous blood sample was drawn from an antecubital vein (1 × 10 ml; BD EDTA vacutainer) for genotypic analysis.

### 

#### Cohort details.

Sixty-five untrained, healthy (identified by physical activity and health questionnaires) female (*n* = 39) and male (*n* = 26) Caucasians (mean ± SD; age = 22.5 ± 4.0 yr; height = 1.71 ± 0.09 m; body mass = 70.9 ± 14.4 kg) were recruited in this population cohort study. All recruits gave written, informed consent to participate in the study, which was approved by the Liverpool John Moores University Ethics Committee in accordance with the Declaration of Helsinki. Inclusion criteria included age between 18 and 35 yr, and potential participants were excluded if they *1*) had performed leg resistance training within the last 6 mo, and *2*) suffered a muscle-tendon injury in the last 12 mo and/or bone fracture in the lower limbs. Participants were instructed to maintain their normal routine, to refrain from drinking alcohol, and to avoid any exercise 48 h before each testing session and throughout the study.

#### Type of study.

Purification of DNA was performed using QIAamp DNA Blood Kit (Qiagen, Crawley, UK) and was prepared according to manufacturer protocols for this candidate SNP study. Each participant was genotyped for *COL1A1* (T > C, rs2249492; G>T rs1800012), *COL2A1* (G>A, rs2070739), and *COL5A1* (T > C, rs12722) SNPs via real-time polymerase chain reaction (PCR) with a Rotor-Gene Q (Rotor-Disk) Instrument (Qiagen). The 10 µl reaction volume included 5 µl Genotyping Master Mix (Applied Biosystems, Foster City, CA), 0.5 µl genotyping assay (Applied Biosystems), 3.5 µl nuclease-free H_2_O (Qiagen), and 1 µl DNA. For control wells, 1 µl nuclease-free H_2_O replaced the DNA template. Reactions were incubated in a 72-well optical plate at 92°C for 15 s (denaturation), followed by annealing and extension at 60°C for 1 min (60 cycles). Each sample was run in duplicate, and there was 100% agreement between samples from the same participant. Genotype calls were performed with Rotor-Gene Q Software 2.3.1 (Qiagen).

#### Details of the SNP(s) studied.

The *COL1A1* rs2249492 and rs1800012 SNPs are located on chromosome 17 at position 50,185,660 and 50,200,388, respectively. The position of the *COL2A1* rs2070739 SNP is at 47,974,193 on chromosome 12, and the *COL5A1* rs12722 SNP is located on chromosome 9 at position 134,842,570 (dbSNP Build 151). The frequency of the effect (major) *COL1A1* rs2249492 T-allele is 0.54; the *COL1A1* rs1800012 G-allele is 0.83; the *COL2A1* rs2070739 G-allele is 0.92; and the *COL5A1* rs12722 T-allele is 0.52. The alleles all refer to the forward DNA strand. Linkage disequilibrium (LD) calculations for the *COL1A1, COL2A1*, and *COL5A1* SNPs were carried out using the LDlink suite and data from the 1000 Genomes Project European ancestry populations (for references, see Appendix 3 in the supplemental material). (The online version of this article contains supplemental material.)

#### Analysis model.

The violations of Hardy-Weinberg equilibrium (HWE) for each SNP were examined using χ^2^ tests. All parameters were normally distributed according to the Shapiro-Wilk test and by inspection of the Q-Q plots. Genotype associations with isometric and isokinetic knee-extension MVC torque, and muscle soreness over time were investigated via two-way mixed ANCOVAs [within-subjects factor: time (pre-, post-, and 48 h post); between-subjects factor: genotype; covariate: sex). Recessive models were used when *n* ≤ 2 for one genotype, and false discovery rate (FDR) was performed to account for multiple testing (FDR set at <20%; for references, see Appendix 3). All results were expressed as means ± SD. MVC data were analyzed with AcqKnowledge software 4.4 (Biopac-Systems), and statistical analyses was performed using SPSS 23 (IBM, Armonk, NY).

## RESULTS

Isometric and isokinetic MVC normalized to body mass, and muscle soreness (all *P* < 0.001) showed a main effect of time, indicating EIMD had occurred. All genotype frequencies for the SNPs were in HWE except for *COL2A1* rs2070739 (χ^2^ = 6.04, *P* = 0.014). There was a low LD for the two *COL1A1* SNPs (D` = 0.736 and R^2^ = 0.077, *P* < 0.001), and for the *COL1A1* and *COL2A1* SNPs with numerous *SGCA* and *TMEM106C* SNPs, respectively. There was a main effect for normalized isometric (rs2249492, *P* = 0.001; rs1800012, *P* = 0.009) and isokinetic (rs2249492, *P* = 0.001; rs1800012, *P* = 0.019) MVC for both *COL1A1* SNPs, with (minor) T-allele carriers (rs1800012) and (major) T-allele homozygotes (rs2249492) being weaker than their respective major (G, rs1800012) and minor (C, rs2249492) allele homozygote counterparts. There was a genotype × time interaction regarding muscle soreness for both *COL2A1* (*P* = 0.002) and *COL5A1* (*P* = 0.004) SNPs. *COL2A1* GG homozygotes showed attenuated soreness post-EIMD compared with A (minor) allele carriers (Supplemental Fig. S1), while *COL5A1* CC (minor allele) homozygotes showed a preferential response compared with *1*) TC genotype post-EIMD and *2*) TT genotype 48 h post-EIMD (Supplemental Fig. S2).

## INTERPRETATION

The T-alleles for both *COL1A1* SNPs were associated with lower maximum strength compared with their respective major (G, rs1800012) and minor (C, rs2249492) alleles. *COL2A1* A (minor) allele carriers and *COL5A1* T (major) allele carriers recovered more slowly from EIMD than their respective *COL2A1* G (major) allele and *COL5A1* C (minor) allele homozygote counterparts. Further work is required to *1*) validate the results in a larger independent cohort and *2*) investigate LD between these SNPs and SNPs of other genes to precisely map the causal variants and genes influencing the phenotypic outcome. However, our results suggest that these four SNPs within three collagen-encoding genes affect muscle strength and recovery following EIMD, possibly by influencing RNA stability, potentially causing dysregulation of the collagen network, thus negatively affecting the mechanical properties of the MTU.

## GRANTS

This study was supported by a Liverpool John Moores University PhD studentship.

## DISCLOSURES

No conflicts of interest, financial or otherwise, are declared by the authors.

## AUTHOR CONTRIBUTIONS

P.B., B.A., J.A.C., V.E., and K.O.J. performed experiments; P.B. and R.M.E. analyzed data; P.B., C.E.S., and R.M.E. interpreted results of experiments; P.B. prepared figures; P.B. drafted manuscript; P.B., M.J.L., B.D., C.E.S., and R.M.E. edited and revised manuscript; P.B., M.J.L., B.D., C.E.S., and R.M.E. approved final version of manuscript; R.M.E. conceived and designed research.

## Supplemental Data

Appendix 3 Appendix 3: List of consortium members and additional references (.docx 20 KB)
