# Supplementary material for: Variations of collagen-encoding genes are associated with exercise-induced muscle damage
Source: Physiol Genomics. 2018 May 25;50(9):691–3. doi: 10.1152/physiolgenomics.00145.2017 (PMC6172608; doi:10.1152/physiolgenomics.00145.2017)
Supplement: Appendix 3 — List of consortium members and additional references (.docx 20 KB) [file Appendix3.docx]

# Appendix 3

**List of the G-REX (Genetics of Recovery and EXercise) consortium members:**

Philipp Baumert^1^, Bethany Adams^1^, Josephine A Cabot^1^, Victoria Edwards^1^, Kelsie O Johnson^1^, Mark Lake^1^, Barry Drust^1^, Claire E Stewart^1^, Robert M Erskine^1,2^

^1^*Research Institute for Sport & Exercise Sciences, Liverpool John Moores University, Liverpool, UK;*

^2^*Institute of Sport, Exercise & Health, University College London, London, UK,*

**Additional References**

6. **Benjamini Y, and Hochberg Y**. Controlling the false discovery rate: a practical and powerful approach to multiple testing. *Journal of the royal statistical society Series B (Methodological)* 289-300, 1995.

7. **Consortium GP**. An integrated map of genetic variation from 1,092 human genomes. *Nature* 491: 56-65, 2012.

8. **Machiela MJ, and Chanock SJ**. LDlink: a web-based application for exploring population-specific haplotype structure and linking correlated alleles of possible functional variants. *Bioinformatics* 31: 3555-3557, 2015.
